# TB/HIV co-infections and associated factors among patients on directly observed treatment short course in Northeastern Ethiopia: a 4 years retrospective study

**DOI:** 10.1186/s13104-015-1664-0

**Published:** 2015-11-11

**Authors:** Daniel Mekonnen, Awoke Derbie, Endalkachew Desalegn

**Affiliations:** Department of Medical Microbiology, Immunology and Parasitology, College of Medicine and Health Sciences, Bahir Dar University, Bahir Dar, Ethiopia; Biotechnology Research Institute, Bahir Dar University, Bahir Dar, Ethiopia; Amhara National Regional State Health Bureau, Research and Technology Transfer Core Process, Bahir Dar, Ethiopia

**Keywords:** TB/HIV co-infection, Associated factors, Ethiopia

## Abstract

**Background:**

Human immunodeficiency virus (HIV) and tuberculosis (TB) are the leading independent global causes of death among patients with infectious diseases. Additionally, due to the shared immune defense mechanisms, they are the leading cause of co-morbidities globally. However, little information was found regarding the proportion of TB/HIV co-infection in the study area. Thus, this study determined the proportion and associated factors of TB/HIV co-infection.

**Methods:**

All TB patients treated from January/2011 to December/2014 were included in this study. Data were collected from three health centers namely; Kobo, Robit and Gobiye. Data were entered, cleared, and analyzed using SPSS version 20. Frequency, percentage, median and range were used to present the data. To assess the associated factors, logistic regression was employed.

**Results:**

Of the total 990 TB patients enrolled in the study, 98.2 % were screened for HIV; of these, 24.3 % were co-infected with TB and HIV. The odds of having TB/HIV co-infection were 3.4 times higher among in the age group of 25–45 years compared to older (≥45 years) age TB patients (OR = 3.4; 95 % CI 2–5). Moreover, the odds of having TB/HIV co-infection were 2.8 and 1.7 times higher among smear positive and smear negative patients with pulmonary TB respectively than patients with extra pulmonary TB. Of 236 co-infected patients, 71.2 % took co-trimoxazole preventive therapy and 76.3 % took antiretroviral treatment.

**Conclusion:**

TB/HIV co-infection is one of the serious public health problems in the study area. Thus, Collaborative TB/HIV activities that reduce the co-morbidities and mortalities should be addressed.

## Background

Human immunodeficiency virus (HIV) and tuberculosis (TB) are the first and second leading causes, respectively of death globally due to a single infectious agent [1]. Due to the shared immune defense mechanisms between the two diseases, TB is a leading preventable cause of death among people living with HIV and vice versa [2–4]. Most of these deaths occur in resource-limited settings [5] like Ethiopia.

According to 2011 Ethiopian demographic health survey, the prevalence of HIV was 1.5 % [6]. Similarly, in urban and rural Amhara region where this study was conducted, the prevalence of HIV was 10.7 and 1.5 %, respectively [7].

The prevalence of TB in Ethiopia was 211/100,000 [8]. Tuberculosis threatens the poorest and most marginalized populations [9, 10]. The resurgence of TB has been fueled by multi factors like HIV and drug resistant TB. And also, the current increasing HIV associated tuberculosis shifted the clinical pattern TB towards smears negative pulmonary (PTB−) and extra pulmonary TB (EPTB) [11, 12].

The prevalence of TB among HIV positive clients in Ethiopia was 7.8 % and in Amhara region, it it was 4.9 % [13]. Conversely, the prevalence of HIV among TB patients in Ethiopia and Amhara region was 20 and 26.5 %, respectively [13].

Information is limited on the proportion of TB, HIV and TB/HIV co-infection in the study area. Also, studies done nationally and in Amhara region could not reflect the real TB/HIV situation in the study area due to methodological variation [13–15]. Unlike others, this study used all TB registers data across 2011–2014 among TB clinics who were treating TB patients from urban and rural area of Kobo and Raya kobo woredas, East of Amhara and/or north eastern Ethiopia to assess the proportion of TB/HIV co-infection and associated factors.

## Methods

### Study setting and period

All patients diagnosed and treated from January/2011 to December/2014 were included in this study. Health centers in Ethiopia in general are a primary health care unit that serves a population of 15,000–25,000 and is comprised of clinics like TB, antiretroviral treatment (ART), maternal and child health, and outpatient. Public health officers and clinical nurses with BSc degree work as practitioner. Moreover, most of laboratory and pharmacy professionals were diploma holder.

Diagnoses of pulmonary TB in the health centers were done using clinical and Ziehl-Neelsen (ZN) microscopy information. Clinical specimen for diagnosis of pulmonary TB was collected using spot-morning-spot sample collection strategy. EPTB cases were diagnosed and referred from nearby Kobo, Woldiya, Dessie, Alamata hospitals and privates clinics. Diagnosis of EPTB was mainly using clinical history with X-ray, ultrasound and/or pathologic techniques. For those diagnosed with active TB, the standard TB treatment regimen, 2(RHZE)/4 (RH) and 2S (HRZE)/1(HRZE)/5(HR)E was started for new and previously treated TB patients, respectively [16]. For HIV screening, nationally approved rapid serological testing algorisms (KHB → StatPak → Uni-gold) were used. As soon as HIV is identified in a TB patient, the patient is enrolled to HIV chronic care and co-trimoxazole preventive therapy (CPT) started [17].

### Operational definitions

#### Cured

A patient with bacteriologically confirmed pulmonary TB at the beginning of treatment who was smear or culture-negative in the last month of treatment.

#### Treatment completed

A patient with TB who completed treatment without evidence of failure, but with no record of sputum smear or culture results, in the last month of treatment.

#### Treatment success (good treatment outcome)

The sum of cured and treatment completed.

#### Died

A TB patient who died from any cause during treatment.

#### Failed

A TB patient whose sputum smear or culture is positive at month 5 or later during treatment.

#### Lost to follow-up

A TB patient who did not start treatment or whose treatment was interrupted for two consecutive months or more.

#### Poor treatment outcome

The sum of treatment failed, died and loss to follow up.

### Inclusion and exclusion criteria

Patients with TB whose record data were missing age, sex and type of TB were excluded from the study. Patients with complete demographic and clinical data (regardless of age groups and sex, type of TB and HIV status) were included in the study.

### Sample size

A total of 990 patients with TB were treated between January/2011 and December/2014. All these patients were included in the study.

### Data collection and tools

Data were collected from three health centers namely Kobo, Robit and Gobiye. Demographic and clinical data such as age, sex, type of TB, HIV, CPT and ART status were retrieved from TB registry.

### Data analysis

All data were entered, cleared, and analyzed using SPSS statistical software package *(IBM Corp. Released 2011. IBM SPSS Statistics for Windows, Version 20.0. Armonk, NY: IBM Corp.).* Frequency, percentage, median and range were used to present the data. To assess the association between dependent and independent variables, logistic regression was used. Associations between variables were determined using odds ratio and 95 % confidence interval (CI). *P* value of <0.05 was considered as statistical significance.

### Ethical considerations

Ethical clearance was obtained from Amhara Regional Ethical Review Committee (RERC) with reference number of HRTT/11/71/2014. In addition, a support letter was written to Zonal Health Department and Kobo and Raya Kobo Woreda Health Offices. Data did not include the patient’s name and kept confidential. The data were used for the purpose of the study only.

## Results

Of the total 990 TB patients enrolled to this study, 551 (55.7 %) were males. The median age of participants were 29 years (range 1–84). The majority of TB patients were in the age groups of 25–45 years. The proportion of EPTB, PTB− and PTB+ were 462 (46.7 %), 332 (33.5 %) and 196 (19.8 %), respectively (Table 1).

**Table 1:** Demographic and tuberculosis profile of TB patients, Northeastern Amhara, Ethiopia, 2015

Variables	Frequency (N)	Percentage (%)
Sex		
Male	551	55.7
Female	439	44.3
Age (years)		
≤24	326	32.9
25–45	458	46.3
≥46	206	20.8
Types of TB		
PTB+	196	19.8
PTB−	332	33.5
EPTB	462	46.7
Treatment outcome		
Poor	96	9.7
Success	853	86.2
Transfer out	30	3
Unknown	11	1.1
HIV status		
HIV positive	236	24.3
HIV negative	736	74.3
Unknown	18	1.8
Total	990	100

Variables	Frequency (N = 236)	Percentage (%)
CPT prophylaxis		
Yes	168	71.2
No	68	28.8
ART status		
On ART	180	76.3
Pre-ART	56	23.6

*ART* antiretroviral therapy, *CPT* co-trimoxazole preventive therapy, *EPTB* extra pulmonary TB, *HIV* human immunodeficiency virus, *PTB−* smears negative pulmonary TB, *PTB+* smear-positive pulmonary TB, *TB* tuberculosis

Among the total TB patients, 972 (98.2 %) were screened for HIV. The remaining 18 (1.8 %) patients had no HIV status data. Of the total HIV screened TB patients, the proportion of TB/HIV co-infection was 236 (24.3 %). The odds of having TB/HIV co-infection was 3.4 times higher in the age group of 25–45 year old as compared to patients with ≥46 years of age (AOR = 3.4; 95 % CI 2–5; P = 0.00). Moreover, the odds of having TB/HIV co-infection were 2.8 and 1.7 times higher among PTB+ and PTB− patients than EPTB, respectively (Table 2). Of that TB/HIV co-infected 71.2 and 76.3 % took CPT and ART, respectively. The proportion of treatment success was 86.2 % (Table 1).

**Table 2:** Regression analysis showing associated factors for TB/HIV co-infection, Northeastern Amhara, Ethiopia, 2015

Predictor variables	TB–HIV confection			COR (95 % CI), P value	AOR (95 % CI), P value
	Yes N (%)	No N (%)	Total N		
Sex					
Female	113 (25.7)	326 (74.3)	439	1.2 (0.9–1.6), 0.2	1.2 (0.9–1.6), 0.17
Male	123 (22.3)	428 (77.7)	551	1	
Age					
≤24	44 (13.5)	282 (86.5)	326	0.9 (0.57–1.6), 0.85	0.9 (0.57–1.6), 0.85
25–45	163 (29.4)	295 (70.6)	418	3.4 (2–5), 0.00	3.4 (2–5), 0.00
≥46	29 (14.1)	177 (85.9)	206	1	1
Types of TB					
PTB+	71 (36.2)	125 (63.8)	196	2.8 (1.9–4), 0.00	2.2 (1.4–3.3), 0.00
PTB−	87 (26.2)	245 (73.8)	332	1.7 (1.2–2.5), 0.002	1.9 (1.3–2.8), 001
EPTB	78 (16.9)	384 (83.1)	462	1	1
TB treatment outcome					
Unknown	4 (36.4)	7 (63.6)	11	2 (0.58–6.98), 0.26	2 (0.56–7.3), 0.23
Transfer out	5 (16.7)	25 (83.3)	30	0.7 (0.26–1.8), 0.48	0.64 (0.23–1.75), 0.38
Poor	39 (40.6)	57 (59.4)	96	2.4 (1.56–3.75), 0.00	2 (1.2–3.1), 0.003
Success	188 (22)	665 (78)	853	1	1

*AOR* adjusted odds ratio, *ART* antiretroviral therapy, *COR* crude odds ratio, *CPT* co-trimoxazole preventive therapy, *EPTB* extra pulmonary TB, *HIV* human immunodeficiency virus, *PTB−* smears negative pulmonary TB, *PTB+* smear-positive pulmonary TB, *TB* tuberculosis

## Discussion

In the present study, 98.1 % of TB patients were screened for HIV which was higher than the latest national data, 71 % [10, 18]. In 2013, 48 % of TB patients globally had a documented HIV test result. In the African Region, 76 % of TB patients knew their HIV status [8]. Among Ethiopian regional states, the highest proportion of people living with HIV/AIDS (PLHIV) was seen in Amhara [7]. This showed that HIV screening practice is better in Amhara region and in the study area in particular. Moreover, it seems that there was better commitment from health professionals and local administrative bodies in terms of screening, communication and social mobilization.

Among those HIV screened TB patients; the proportion of TB/HIV co-infection was 24.3 % which was much higher than reports from Ethiopia, 6.3–20 % [10, 13, 16, 17, 19]. It was also higher when compared to a similar study in Nigeria, 20.5 % [20]. Overlapping co-morbid diseases are growing in resource-limited countries like Ethiopia [11]. This might be due multi-TB and HIV related risk factors. First, it is well known that, people in this area have the practice of dating sisters-in-law or brothers-in-law [observation]. This practice would have a significant impact on HIV transmission. Second, the community especially farmers in the study area are well known drinkers of local beer (Teji in local Amharic Language), this action might push people to have unprotected sexual practice. Third, the area is one of draughts prone and food unsecured area [21]. Thus, there are many migrants to Middle East for home maid and daily labor after having divorced. Moreover, so many beautiful ladies enforced to sex work due to financial constraints. Furthermore, the associations between poverty and TB have been known [9, 10, 22–24]. This might increase TB/HIV co-infection cases in the area. Fourth, the presence of many illegal private TB drug sellers in the area might contributed for high TB and drug resistant TB. Lastly, high number of HIV means, there would be many HIV associated tuberculosis. All these factors might have direct or indirect contribution to the high TB/HIV co-infection rate. To identify which factors are more important than other, population based study must be done.

In contrast, our TB/HIV proportion data was lower than studies done in Debre markos 44 % [14], Dabat-Gondar 34 % [15] and Kenya 41.8 % [25]. This might be due to differences in the study site. These studies were conducted at referral and teaching hospitals where many chronic, end stage diseases and referral cases were seen. Our study was conducted in health centers. Globally, 13–14.8 % of people who developed TB are HIV positive [7, 9, 26, 27]. Prevalence of TB/HIV co-infection was 31.25 % in African countries [7, 28]. In 2012, the prevalence of TB/HIV co-infection was 43 % in Africa [9] and as high as 50–80 % in parts of sub-Saharan Africa [27].

The ART coverage in the present study was higher, 76.3 %, as compared to a WHO report from Ethiopia, 68 %, but lowers than WHO target of 100 % [10, 18]. Other reports showed that ART coverage was 50.5 and 40.2 % in Ethiopia and Amhara region [13], respectively. This might be due to proximity of health centers to the community. Moreover, communication and social mobilization might be done by local stake holders to diagnose and linked more patients to ART. Furthermore, according to unpublished reports from the community, the practice of safe sex and use of condoms is low but people’s awareness of the value of ART is high.

The proportion of TB/HIV co-infection among women was relatively higher in the present study (Table 2). This corroborates WHO reports. More young women notified with TB/HIV than young men in Africa [29–31]. Out of the 346 TB cases in Kenya, 41.8 % of were co-infected and female to male TB/HIV co-infection prevalence ratio was 1.35:1 [25]. The possible reason might be due to socio economic factors and health seeking behavior of women. As compared to men, women have no control of financial resources at household levels. Moreover, there is higher rate of transient and permanent immune suppression with pregnancy, lactation and HIV in women than men [29].

In the present study, TB/HIV co-infection was significantly associated with age group of 25–45 years and PTB+, P < 0.05 (Table 2). It was well known that TB and HIV affect reproductive age groups of the population [8]. In contrast to the literature evidence, PTB+ was significantly associated with HIV than PTB− and EPTB in the present study. This might be due to multi factors. First, the relatively higher ART and CPT coverage was found in the study area. If PTB+/HIV co-infected people adhered well on ART and CPT, they would have high level of innate and cell mediated immunity. Thus, these people would have high probability for being smear positive. Second, it is known that EPTB and PTB− are HIV associated than PTB+ [12]. However, this study could not differentiated HIV associated TB from non HIV associated TB. Thus, if TB infection and diagnosis occurred before HIV infection, the proportion of PTB+/HIV would be higher than EPTB/HIV and PTB−/HIV co-infection. The third, possible reason might be due to, over-diagnosis of EPTB and PTB− cases. Because, higher proportion of EPTB and PTB− cases were diagnosed and transferred in from private clinics which did not have any imaging and pathologic techniques. This justification was supported by, health professionals in the study area (expert opinion). Additionally, Ehlers et al. reported that only 2 of the 76 PTB− patients had been diagnosed correctly [32]. Moreover, a study by Iwnetu et al. concluding that up to 15 % of all EPTB cases could be wrongly diagnosed [33]. The last possible reason might be due to zoonotic transmission of TB and genetic features of the pathogen and/or the host population. This was in line with the latest report by Berg et al. According to this report, having regular and direct contact with live animals, was a significant risk factor for lymph node (TBLN) when compared to pulmonary TB. In addition, no association was found with HIV infection [34]. The study area is known with large live stock populations, sharing of shelter between human and livestock, use of raw meat and milk.

TB/HIV co-infection was significantly associated with poor TB treatment outcome in the present study (AOR: 2; 95 % CI 1.2–3.1; P = 0.003) (Table 2). This might be due to the presence of TB and HIV drug interaction that make low adherence to anti-TB drugs. Tuberculosis case-fatality rates in Africa were 16–35 % among HIV positives and 4–9 % in HIV-negative patients [35, 36]. This indicates that full DOTS implementation alone is insufficient to control TB. Thus, collaborative TB/HIV activities aimed to reduce the burden of diseases are very important [37].

## Conclusion and recommendations

The proportion of TB/HIV co-infections were higher in the study area which indicates that people’s awareness on transmission and control mechanism of TB and HIV is low. On the other hand, 98.1 % of TB patients were screened for HIV but ART coverage was 76.3 % which was lower than WHO target of 100 %. Moreover, proportion of TB/HIV co-infection was associated with women, age group of 25–45 years, PTB+ and poor TB treatment outcome. Hence, sex and age targeted interventions like awareness creation training should be given to female and sexually active age (25–45 years) groups of the population. Moreover, communication and social mobilization must further strengthen to achieve 100 % ART coverage. TB/HIV co-infected people were found to be more infectious. Thus, the laboratory professionals’ capacity must be strengthen to detect those infectious cases. Additionally, diagnostic quality of EPTB and PTB− must strengthen by implementing rapid and sensitive diagnostic technologies like, Fluorescent Microscopy, Xpert MTB/Rif. Furthermore, to have good TB treatment outcome, prompt and community involved DOTS based anti-TB treatment should be in place.

## Abbreviations

AIDS: acquired immunodeficiency syndrome
AOR: adjusted odds ratio
ART: antiretroviral therapy
CI: confidence interval
COR: crude odds ratio
CPT: co-trimoxazole preventive therapy
DOTS: directly observed treatment for short course
E: ethambutol
EPTB: extra pulmonary TB
H: isoniazid
HIV: human immunodeficiency virus
ICF: intensified case-finding of TB
IPT: isoniazid preventive therapy
PLHIV: people living with HIV
PTB−: smears negative pulmonary TB
PTB+: smear-positive pulmonary TB
R: rifampicin
RERC: Regional Ethical Review Committee
S: streptomycin
SPSS: statistical package social sciences
TB: tuberculosis
TBLN: lymph node TB
WHO: World Health Organization
Z: pyrazinamide
ZN: Ziehl-Neelsen
